# Supplementary material for: Are Gait Patterns during In-Lab Running Representative of Gait Patterns during Real-World Training? An Experimental Study
Source: Sensors (Basel). 2024 May 1;24(9):2892. doi: 10.3390/s24092892 (PMC11086149; doi:10.3390/s24092892)
Supplement: Supplementary file 1 [file sensors-24-02892-s001.zip › sensors-2890698-supplementary.pdf]

Supplementary Figures & Tables

Table S1. Validation analysis of gait metrics. Table shows repeated measures Bland-Altman gait metric mean biases, upper and lower limits of agreement (LoA), mean subject-wise correlation coefficient (*r*), and standard deviation (SD) of subject-wise correlation coefficient.

**Sensor-measured gait metric**	**Bias**	**Lower LoA**	**Upper LoA**	**Mean *r***	***r* SD**
Foot pod: speed (m/s)	0.07	-0.06	0.21	>0.99	<0.01
Chest strap: step length (mm)	25.36	-24.62	75.33	>0.99	<0.01
Foot pod: stance time (ms)	0.68	-33.00	34.36	0.97	0.02
Chest strap: vertical ratio (%)	2.22*	0.32	4.13	0.97	0.02
Chest strap: stance time (ms)	-9.70	-42.16	22.76	0.94	0.08
Chest strap: cadence (spm)	-0.05	-1.79	1.69	0.93	0.08
Foot pod: cadence (spm)	-0.11	-2.00	1.78	0.92	0.09
Chest strap: stance time percent (%)	-1.28	-5.78	3.21	0.83	0.16
Chest strap: vertical oscillation (cm)	2.67*	0.76	4.58	0.63	0.19
Foot pod: vertical oscillation (cm)	0.19	-2.00	2.37	0.31	0.32
Foot pod: leg stiffness (kN/m)	-1.56	-4.63	1.52	0.15	0.43
Chest strap: stance time balance (%)	-0.24	-2.38	1.90	0.12	0.15

*Statistically significant bias (limits of agreement exclude zero)

Table S2. Treadmill run protocol. Participants were randomly assigned to one of four run protocols, with the two slowest speeds at the beginning, a block of random speeds in the middle, and the two fastest speeds at the end, each preceded by easy recovery. Running speeds were relative to the participant's preferred running speed (PS) for a typical training session.

Time	Protocol A	Protocol B	Protocol C	Protocol D
0:00-5:00	Instructions and gait system synchronization →			
5:00-6:30	1.12 m/s (walk warm-up)* →			
6:30-8:00	1.56 m/s (walk warm-up)* →			
8:00-11:00	70% PS →			
11:00-14:00	75% PS →			
14:00-17:00	90% PS	105% PS	85% PS	110% PS
17:00-20:00	85% PS	90% PS	80% PS	95% PS
20:00-23:00	105% PS	85% PS	90% PS	100% PS
23:00-26:00	95% PS	110% PS	95% PS	85% PS
26:00-29:00	115% PS	100% PS	115% PS	90% PS
29:00-32:00	80% PS	80% PS	100% PS	115% PS
32:00-35:00	100% PS	95% PS	110% PS	105% PS
35:00-38:00	110% PS	115% PS	105% PS	80% PS
38:00-40:00	80% PS →			
40:00-42:00	120% PS →			
42:00-44:00	80% PS →			
44:00-46:00	125% PS →			
46:00-48:00	1.12 m/s (walk cool-down)* →			

*Walking trials not included in present analysis
